# An Evaluation of Common Carotid Artery Intima-Media Thickness in Patients With Acute Ischemic Stroke: A Case-Control Study

**DOI:** 10.7759/cureus.101287

**Published:** 2026-01-11

**Authors:** Koshy T Sam, Jayashankar CA, Venkataramana Kandi, Ganaraja V Harikrishna, Venkata Bharat Kumar Pinnelli, Manish GR, Snigdha Reddy, Srilekha N, Koshy T Mathew, Atul S Sucharitha

**Affiliations:** 1 Internal Medicine, Vydehi Institute of Medical Sciences and Research Centre, Bengaluru, IND; 2 Clinical Microbiology, Prathima Institute of Medical Sciences, Nagunur, IND; 3 Neurology, National Institute of Mental Health and Neuro Sciences, Bengaluru, IND; 4 Biochemistry, Vydehi Institute of Medical Sciences and Research Centre, Bengaluru, IND; 5 General Medicine, Vydehi Institute of Medical Sciences and Research Centre, Bengaluru, IND; 6 General Medicine, Malankara Orthodox Syrian Church Medical College, Kochi, IND

**Keywords:** acute ischemic stroke, cerebrovascular, common carotid artery intima-media thickness, noninvasive, stroke

## Abstract

Introduction: Stroke has major clinical and economic ramifications and is one of the world's top causes of morbidity and mortality. Over the past years, there has been an increasing trend in the occurrence of stroke in India. The association between increased common carotid artery intima-media thickness (CIMT) and stroke risk in India's general population has not been well studied. Thus, the purpose of this study is to evaluate, compare, and establish a relationship between the CIMT in acute ischemic stroke (AIS) patients and healthy individuals.

Methods: This prospective, observational, cross-sectional, and case-control study was conducted among patients admitted to the Departments of General Medicine and Neurology at a tertiary-care teaching hospital in Bengaluru, India. The study was conducted between March 2023 and March 2025 and included 50 patients. A total of 25 patients with AIS were included as cases, and 25 healthy individuals were included as controls. The carotid artery Doppler test was used to measure the CIMT. The hematological and biochemical parameters of all the study participants were measured using standard laboratory techniques.

Results: In both groups, the majority of individuals were aged 50 years or older. The control group's mean age was 56.64 ± 10.03 years, compared to 54.40 ± 12.36 years in the case group (t = 0.70, p = 0.48). In contrast to the control group, which had 92% (23) male and 8% (two) female participants (chi-square (𝜒^2^) = 0.50, p = 0.22), 80% (20) of the cases were male and 20% (five) were female participants. AIS patients had a considerably higher mean CMIT (1.11 ± 0.22 mm), which was significantly (t = -3.612, p = 0.0007) greater than controls (0.91 ± 0.19 mm). The comparison of hematological and biochemical parameters, including the lipid profile, between the case and control groups revealed statistically significant differences in total leucocyte count (TLC) and hemoglobin (Hb) levels. The mean TLC was significantly lower (t = 5.51 and p < 0.0001) in cases (8.89 ± 2.58 cells/mm^3^) than in controls (12.78 ± 2.41 cells/mm^3^). Similarly, Hb was significantly higher (t = -6.51 and p < 0.0001) in cases (13.17 ± 2.47 g/dL) than in controls (8.49 ± 2.61 g/dL). Among the lipid parameters, only low-density lipoproteins demonstrated borderline significance (p = 0.05) with CIMT.

Conclusions: Patients with AIS have considerably higher CIMT, which supports its application as a noninvasive and affordable diagnostic modality for identifying people who are more likely to experience cerebrovascular episodes.

## Introduction

Stroke has major clinical and economic ramifications and is one of the world's top causes of death and morbidity. It is a neurological condition that arises when blood flow to the brain is disrupted [1]. Also referred to as a cerebrovascular disease, stroke is defined by the abrupt onset of a neurological deficiency brought on by a particular vascular event [2]. These deficiencies are usually limited to a specific area of the brain and are frequently associated with reduced blood flow to that location [3]. Ischemic stroke and hemorrhagic stroke are the two primary categories of stroke. The majority of cerebrovascular events are caused by ischemia, with the remaining cases being caused by primary cerebral bleeding, including intracerebral hemorrhage (ICH) and subarachnoid hemorrhage (SAH). Different kinds of cerebrovascular accidents are associated with a number of significant risk factors, such as smoking, obesity, hypercholesterolemia, type 2 diabetes mellitus (T2DM), and systemic hypertension (HTN). Carotid artery atherosclerosis (ATS) is one of the most important risk factors for cerebrovascular disease, and it contributes significantly to the mechanism of stroke in over one-third of patients [4].

According to predictions from 2020, there were 89.13 million cases of all stroke subtypes worldwide, with 68.16 million cases of acute ischemic stroke (AIS). There were 18.88 million cases of ICH and 8.09 million cases of SAH [5]. Globally, there were 7.08 million stroke-related deaths, of which 3.48 million were from ischemic stroke, 3.25 million from ICH, and 0.35 million from SAH. Over the past 17 years, there has been a sharp increase in the occurrence of stroke in India, rising by almost 50%. According to current estimates, one in four people may have a stroke at some point in their lives [6]. The chance of having a stroke after 55 years is one in five for women and one in six for men, indicating that the risk increases dramatically with age. India has a wide range of stroke prevalence rates, from 44.54 to 150 per 100,000. These figures highlight the growing prevalence of stroke and the pressing need for efficient management and preventative techniques. According to a thorough Global Burden of Disease research carried out in India, stroke is a serious public health issue that causes 9.4 million fatalities [7]. With 28.5 million Disability-Adjusted Life Years lost as a result of stroke, the study also highlighted the substantial impact of the condition on general health and productivity.

Additional diagnostic tests are advised by current clinical guidelines to assess vascular problems prior to or following any cerebrovascular incident. The use of carotid artery duplex ultrasonography (USG) to evaluate the cerebral vasculature's thickness and smoothness is growing [8]. Measuring common carotid artery intima-media thickness (CIMT), a marker of ATS and a potential risk factor for stroke, is a primary diagnostic technique for assessing ATS diagnosis and prognosis [9]. Furthermore, deep learning and machine learning technologies based on artificial intelligence have become more popular in the diagnosis and prognosis of AIS and other clinical disorders. This further supports the necessity for an increasing amount of CIMT data that might establish common metrics in both healthy and diseased persons relevant to various geographic locations and sociocultural contexts, allowing clinicians to anticipate, diagnose, and treat patients [10,11]. The association between increased CIMT and stroke risk in India's general population has not been well studied. Therefore, the aim of this study was to assess, compare, and determine a correlation between the common CIMT in AIS patients and healthy individuals.

## Materials and methods

Patients admitted to the Departments of General Medicine and Neurology at the tertiary-care teaching hospital in Bengaluru, India, participated in this prospective, observational, case-control study. The trial included 50 patients and was conducted between March 2023 and March 2025. There were 25 cases of AIS and 25 healthy controls among the 50 recruited study participants. All individuals who met the inclusion criteria were enrolled after giving their informed consent, and the Vydehi Institutional Ethics Committee, Bengaluru, India, granted ethics approval (VIEC/2023/APP/PG/086).

Sample size estimation

To compare two proportions in a case-control study, the sample size was calculated using the Fleiss community correction. "Increased CIMT" was identified as the risk factor. The sample size (n) was estimated using the formula: $n = \frac{r}{r+1} \cdot \frac{(Z_{1-\alpha/2} + Z_{1-\beta})^2}{(p_1 - p_2)^2 \, \tilde{p}(1-\tilde{p})}$. With a control-to-case ratio (r) of 1, the anticipated percentage in cases (p1) was 48.33, and in the control (p2) was 3.33. The computation was done with 80% power ((Z1 - β = 0.84) and a 95% confidence level (Z1 - α/2 = 1.96)). With a total of 36 individuals, the necessary sample size was determined to be 18 for the exposed group and 18 for the control group. To boost the study's power, a final sample size of 50 was chosen.

Inclusion criteria

The study included individuals admitted to the Departments of General Medicine and Neurology for treatment of AIS, as well as individuals aged 18-80 who voluntarily agreed to participate. The control group consisted of healthy people who visited the hospital for routine checkups.

Exclusion criteria

The study excluded individuals with valvular heart disease, thrombophilia disorders, a history of stroke lasting longer than two weeks, and those receiving statin medication for more than a year for whatever reason.

Laboratory investigations

Computed tomography and magnetic resonance imaging of the brain were used to diagnose AIS, and carotid artery Doppler was used to measure the CIMT using A and B-mode USG images of the common carotid artery and to measure the thickness of the two inner layers of the artery wall. Additionally, all study participants had their hematological and biochemical parameters analyzed, which included measurements of complete blood count, random blood glucose (RBG), glycated hemoglobin (HbA1c), blood urea, serum creatinine, and serum lipid profile consisting of high-density lipoproteins (HDL), low-density lipoproteins (LDL), very low-density lipoproteins (VLDL), and triglyceride (TG). All the parameters were measured using standard laboratory techniques. The glucose oxidase-peroxidase technique was utilized to determine RBG. Glycerol phosphate oxidase was used to measure serum TG. Friedewald's equation and the direct enzymatic approach were used to estimate HDL, LDL, and VLDL cholesterol levels [12,13]. The hematological and biochemical parameters were analyzed using Sysmex XN-1000™ Automated Hematology Analyzer (Sysmex Corporation, Kobe, Japan) and VITROS® 5600 Integrated System (QuidelOrtho Corporation, San Diego, CA) for clinical chemistry and immunoassay testing.

Protocols followed in CIMT measurement

Artery Parameters

Every patient had both sides of the CIMT measurement performed using the A- and B-mode USG probe with a frequency ranging from 2.0-14.0 2 MHz; the data provided per patient are the thickest area obtained from both carotid arteries (either the left or the right, whichever is thickest); it is not the average of both sides, but rather the single thickest value obtained; there is no point of maximum thickness because it varies from patient to patient.

Operator Parameters

Every measurement was taken by two doctors. Postgraduate residents in the hospital's radiology departments took the initial measurements. Assistant professors and/or higher cadre consultants in the hospital's radiology departments confirmed all of these measurements. To avoid interobserver variability, each measurement was confirmed by two medical experts.

Statistical analysis

Data entry was performed using Microsoft Office 2019 Excel (Microsoft Corp., Redmond, WA), and statistical analysis was conducted using IBM Statistical Package for the Social Sciences Statistics for Windows, version 20 (IBM Corp., Armonk, NY; released 2011). Categorical data were summarized using frequencies and percentages, and the chi-square (𝜒^2^) test was performed to compare groups. Group mean differences were assessed using the independent t-test, and continuous variables were shown as mean and standard deviation. A p value of less than 0.05 was considered statistically significant.

## Results

The age distribution of patients and controls showed that most participants were older than 50 years. The mean age of the case group was 54.40 ± 12.36 years, compared with 56.64 ± 10.03 years in the control group. An independent t-test was used in the statistical analysis, which revealed no significant difference in age between the two groups (t = 0.70, p = 0.48). In contrast to the control group, which comprised 92% (23) male and 8% (two) female participants, the cases comprised 80% (20) male and 20% (five) female participants. There was no statistically significant variation in the distribution of genders between the two groups (𝜒^2^ = 0.50, p = 0.22). The detailed age-wise distribution of cases and controls is shown in Table 1.

**Table 1:** Age-wise distribution of cases and controls p value is calculated from the mean and SD of cases and controls A p value of less than 0.05 was considered statistically significant SD: standard deviation

Age (years)	Cases, n (%)	Controls, n (%)	p value
18-30	1 (4)	0 (0)	Student’s t-test = 0.70, p value = 0.48
31-40	4 (16)	2 (8)	
41-50	2 (8)	5 (20)	
51-60	9 (36)	7 (28)	
>60	9 (36)	11 (44)	
Total	25 (100)	25 (100)	
Mean ± SD	54.40 ± 12.36	56.64 ± 10.03	

**Figure 1:**
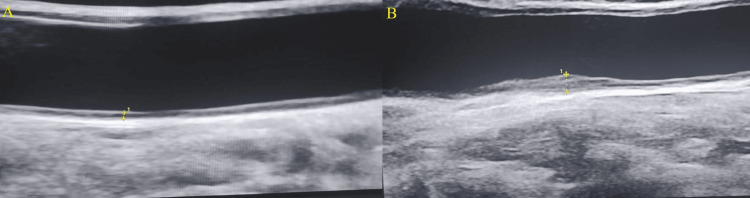
A sample USG image showing CIMT measured in control and case (A) Control: 53-year-old male patient (yellow markings depicting the CIMT measured at 0.30 mm). (B) Case: 43-year-old male patient (yellow markings depicting CIMT measured at 1.80 mm) USG: ultrasonography; CIMT: common carotid artery intima-media thickness

The two groups' CIMTs differed significantly from one another. AIS patients had a significantly greater (t = -3.44, p = 0.0012) mean CIMT (1.11 ± 0.22 mm) than controls (0.91 ± 0.19 mm) (Figure 1).

The CIMT was assessed across several age groups in the patients and controls. The average CIMT values in cases were 1.00 ± 0.00 mm for individuals aged 18-30, 1.04 ± 0.11 mm for those aged 31-40, 1.05 ± 0.07 mm for those aged 41-50, 1.06 ± 0.31 mm for those aged 51-60, and 1.21 ± 0.18 mm for those aged beyond 60. The case group's total mean CIMT was 1.12 ± 0.22 mm. Despite a progressive increase in CIMT with age, the difference across age groups was not statistically significant (p = 0.596). This demonstrated that there was no significant age-related variation in CIMT among AIS patients in this study cohort. In the control group, no participants were present in the 18-30 years age group. The mean CIMT values observed were 0.80 ± 0.28 mm for the 31-40 years group, 0.96 ± 0.21 mm for the 41-50 years group, 0.98 ± 0.19 mm for the 51-60 years group, and 0.86 ± 0.18 mm for those above 60 years of age. The overall mean CIMT in the control group was 0.91 ± 0.19 mm. A significant variation in CIMT was observed across the 31-40 years (p = 0.0002), 41-50 years (p = 0.0476), and > 60 years (p < 0.0001) groups in both cases and controls. Moreover, the overall difference was statistically significant (p = 0.0007). This indicated that age had a significant effect on CIMT in individuals with AIS and healthy controls in this study (Table 2).

**Table 2:** Age-wise comparison of carotid artery intima-media thickness in cases and controls A p value of less than 0.05 was considered statistically significant CIMT: common carotid artery intima-media thickness; SD: standard deviation; NA: not applicable

Study group age (years)	CIMT cases (mm), n = 25, mean ± SD	CIMT controls (mm), n = 25, mean ± SD	Student’s t-test	p value
18-30	1.00 ± 0.00	0	NA	NA
31-40	1.04 ± 0.11	0.8 ± 0.28	-3.989	0.0002
41-50	1.05 ± 0.07	0.96 ± 0.21	-2.033	0.0476
51-60	1.06 ± 0.31	0.98 ± 0.19	-1.100	0.2768
>60	1.21 ± 0.18	0.86 ± 0.18	-6.875	<0.0001
Total	1.12 ± 0.22	0.91 ± 0.19	-3.612	0.0007

A gender-based comparison of CIMT between controls and cases was conducted. For male patients, the average CIMT was 1.08 ± 0.23 mm; for female patients, it was 1.22 ± 0.19 mm. Despite not being statistically significant (p = 0.22), the mean CIMT for female patients was somewhat greater than that of male patients. This suggests that the CIMT in AIS patients was not considerably impacted by gender. The controls had an average CIMT of 0.75 ± 0.35 mm in women and 0.93 ± 0.18 mm in men. Although the difference was not statistically significant (p = 0.21), the mean CIMT in male participants was higher than in female participants. These findings demonstrated that gender had no appreciable effect on CIMT in those without AIS (Table 3).

**Table 3:** Gender-wise comparison of carotid artery intima-media thickness in cases and controls A p value of less than 0.05 was considered statistically significant CIMT: common carotid artery intima-media thickness; SD: standard deviation

Study group	CIMT (mm) in men, mean ± SD	CIMT (mm) in women, mean ± SD	Student’s t-test	p value
Cases (n = 25)	1.08 ± 0.23 (n = 20)	1.22 ± 0.19 (n = 5)	1.252	0.22
Controls (n = 25)	0.93 ± 0.18 (n = 23)	0.75 ± 0.35 (n = 2)	-1.281	0.21

The comparison of hematological and biochemical parameters, including the lipid profile, between the case and control groups revealed statistically significant differences in TLC and Hb levels. The mean TLC was significantly lower in cases (8.89 ± 2.58 cells/mm^3^) compared to controls (12.78 ± 2.41 cells/mm^3^) (t = 5.51 and p < 0.0001). Similarly, Hb was significantly higher in cases (13.17 ± 2.47 g/dL) than in controls (8.49 ± 2.61 g/dL) (t = -6.51 and p < 0.0001). Other parameters, including platelet count, RBG, HbA1c, blood urea, serum creatinine, HDL, LDL, VLDL, and TG levels, showed no statistically significant difference between the two groups, although LDL levels revealed borderline significance (p = 0.05) (Table 4).

**Table 4:** Comparison of hematological and biochemical parameters in cases and controls A p value of less than 0.05 was considered statistically significant TLC: total leukocyte count; RBG: random blood glucose; HbA1c: glycated hemoglobin; HDL: high-density lipoprotein; LDL: low-density lipoprotein; VLDL: very low-density lipoprotein; SD: standard deviation

Parameters	Reference range	Case (n = 25), mean ± SD	Control (n = 25), mean ± SD	Student’s t-test	p value
TLC (cells/mm^3^)	4,000-11,000	8.89 ± 2.58	12.78 ± 2.41	5.51	<0.0001
Hemoglobin (g/dL)	Males: 12.3-17; Females: 9.9-14.3	13.17 ± 2.47	8.49 ± 2.61	-6.51	<0.0001
Platelets (cells/mm^3^)	150,000-400,000	254.72 ± 52.14	241.12 ± 77.67	-0.72	0.47
RBG (mg/dL)	<150	167.16 ± 72.10	144.25 ± 63.76	-1.19	0.24
HbA1c (%)	<6	7.54 ± 2.98	7.68 ± 2.29	1.19	0.85
Blood urea (mg/dL)	15-40	36.05 ± 28.77	31.16 ± 16.50	-0.74	0.46
Creatinine (mg/dL)	Males: 0.7-1.4; Females: 0.6-1.2	1.45 ± 1.92	1.07 ± 0.56	-0.95	0.35
HDL (mg/dL)	≥60	37.77 ± 6.36	38 ± 10.31	0.08	0.92
LDL (mg/dL)	<100	109.78 ± 38.91	88.30 ± 37.85	-1.98	0.05
VLDL (mg/dL)	<30	32.29 ± 12.39	27.67 ± 11.43	-1.37	0.18
Triglyceride (mg/dL)	<150	171.85 ± 60.56	144.87 ± 54.21	-1.66	0.10

## Discussion

The etiology of most ischemic strokes is based on ATS, a persistent inflammatory condition that affects the artery wall. Modifiable risk factors such as HTN, diabetes mellitus, smoking, and hyperlipidemia, as well as nonmodifiable factors such as age and heredity, all play important roles in stroke risk. While standard scoring systems help to identify high-risk individuals, their limitations necessitate the use of additional, more objective markers. CIMT has emerged as a noninvasive, reliable marker for assessing ATS and predicting cerebrovascular and cardiovascular events. The present study aimed to compare CIMT between patients with AIS and healthy controls.

The study found no significant difference in mean age between AIS cases and controls (54.40 ± 12.36 vs. 56.64 ± 10.03 years; p = 0.48). Danuaji et al. [14] showed similar age distribution trends, with both the case and control groups having a rather uniform dispersion over the age categories 30-45 and 46-60 years. Observations made by Moghtaderi et al. reported a higher average age of 62 ± 11.7 years for their study population, which included patients with ischemic stroke and ICH [15].

Male patients made up 80% of the case group and 92% of the control group in the current investigation. In the case and control groups, the proportion of female patients was 20% and 8%, respectively. The difference in gender distribution was not statistically significant (χ^2^ = 0.50, p = 0.22), despite the numerical difference. Previous studies have revealed similar gender patterns. For example, Danuaji et al. [14] found that the gender distribution was more evenly distributed, with 55.69% male and 44.31% female patients in the stroke group and 58.22% male and 41.78% female patients in the controls. These results demonstrate the predominance of men among stroke patients. The finding that men are often more impacted by ischemic stroke is further supported by Jain et al.'s [16] findings that 61% of their stroke patients were men. In addition to any biological and behavioral variations affecting cardiovascular risk, this tendency may be explained by a higher incidence of modifiable risk factors among men, such as alcohol intake, smoking, and HTN.

A statistically significant difference was seen in the CIMT between AIS patients and healthy controls in the current investigation. The AIS group's mean CIMT (1.11 ± 0.22 mm) was significantly (t = -3.612, p = 0.0007) greater than the control group's (0.91 ± 0.19 mm). The idea that subclinical ATS, as indicated by elevated CIMT, plays a critical role in the pathophysiology of ischemic stroke is supported by the notable rise in CIMT among stroke patients. In a research study from Bengaluru, which included 50 patients and 50 controls, 45 (90%) controls had CIMT between 0.02 and 0.60 mm. The majority of AIS patients had CIMT values greater than 0.60 mm. Furthermore, the findings demonstrated that ischemic cerebrovascular accidents are more likely in those with risk factors and CIMT ≥0.60 mm [17]. These results are in line with an earlier study by Ahuja et al. [18], which also showed that patients with AIS had considerably higher CIMTs than healthy people. This demonstrates the value of CIMT as a trustworthy and noninvasive surrogate indicator of ATS.

​In contrast to the current study's findings, a similar study conducted among AIS patients in Puducherry, southern India, reported lower mean CIMT values in both the patient (0.79 mm) and control (0.60 mm) groups [19]. A study in West Bengal included 100 AIS patients and 50 healthy controls, with a mean age of 57.21 ± 7.71 years in cases and 54.76 ± 7.49 years in controls. The study found that cases had a CIMT of 0.84 ± 0.19 mm, whereas controls had a CIMT of 0.60 ± 0.09 mm (p < 0.001) [20]. In contrast, a previous study conducted in Kerala, in southern India, reported a mean CIMT of 0.91 ± 0.12 mm in AIS cases and 0.79 ± 0.13 mm in controls (p < 0.001) [21]. Another study from Uttarakhand, in northern India, reported a mean left CIMT of 0.73 ± 0.29 mm in cases and 0.57 ± 0.14 mm in controls [22]. In a multicentric study, conducted in New Delhi, the capital state of India, that included 1,229 people aged ≥30 years and who had no previous cardiovascular disease history, the mean CIMT assessment revealed men (0.60 ± 0.12 mm) had significantly (p < 0.001) higher CIMT compared with women (0.579 ± 0.11 mm) [23]. The CIMT measurements for the right were 0.49 mm, and for the left 0.50 mm, in a study conducted among a normotensive population aged 40-55 in Jharkhand, eastern India [24]. A recent multicenter study that explored the relationship of AIS with CIMT assessed the factors that potentially contributed to the development of AIS. The results of this study revealed that AIS patients with predisposing factors had higher CIMT (≥0.90 mm) than those without predisposing factors (<0.90 mm) [25]. A study of 200 HTN patients included 100 stroke patients (53.75 ± 15.30 years) and 100 nonstroke patients (53.98 ± 15.94 years), with male patients accounting for 54% of the stroke patients. CIMT was substantially greater in ischemic stroke patients (1.03 ± 0.11 mm) than in hemorrhagic stroke patients (0.98 ± 0.13 mm; p = 0.040). The study indicated that 78 individuals with HTN, T2DM, and dyslipidemia had CIMT <0.90 mm, 91 had CIMT between 0.90 and 1.10 mm, and 31 had CIMT >1.10 mm (Table 5) [26].

**Table 5:** The common carotid artery intima-media thickness in ischemic stroke and control persons reported across India A p value of less than 0.05 was considered statistically significant AIS: acute ischemic stroke; CIMT: common carotid artery intima-media thickness; HTN: hypertension; T2DM: type 2 diabetes mellitus; CVD: cardiovascular disease; TC: total cholesterol; LDL: low-density lipoproteins; TG: triglycerides; HbA1c: glycated hemoglobin; FBG: fasting blood glucose

Study	Year and state	Patient group, age, and sex	CIMT in cases	CIMT in controls (mm)	Significance/p value
This study	2025, Telangana	25 AIS patients aged 54.40 ± 12.36 years and 25 healthy controls aged 56.64 ± 10.03 years	1.11 ± 0.22 mm	0.91 ± 0.19 mm	0.0007
Vishwakarma et al. [26]	2025, Uttar Pradesh	200 ischemic stroke patients aged 53.75 ± 15.30 years, with 54% males, and a nonstroke group aged 53.98 ± 15.94 years	78 patients with HTN, T2DM, and dyslipidemia had <0.9 mm; 91 patients with HTN, T2DM, and dyslipidemia had 0.90-1.10 mm; 31 patients with HTN, T2DM, and dyslipidemia had >1.10 mm	No specific data provided	CIMT was significantly higher in ischemic stroke patients (1.03 ± 0.11 mm) compared to hemorrhagic stroke patients (0.98 ± 0.13 mm; p = 0.040); CIMT >0.9 mm in TC (p = 0.0022), TG (p = 0.018), LDL (p = 0.024), FBG (p = 0.015), and HbA1c >10% (p = 0.022)
Nair et al. [25]	2024, multicenter study: Tamil Nadu, Karnataka, Andhra Pradesh, Maharashtra	60 AIS patients including 43 (71.67%) males, 18-30 (2, 3.3%), 31-40 (3, 5%), 41-50 (12, 20%), 51-60 (14, 23.3%), 61-70 (18, 30%), 71-80 (11, 18.3%)	≥0.90 mm among patients with predisposing factors like hypertension. <0.9 mm among patients without risk factors	Not included	Smoking and hypertension (p = 0.0005), and males (p = 0.03) had CIMT >0.90 mm
Kaur et al. [24]	2024, Jharkhand	100 hypertensive and 100 normotensive individuals aged 40-55 years	0.97 mm	0.49 mm	<0.001
Ravikanth [21]	2019, Kerala	500 AIS patients, mean age 56.40 years in cases and 55.20 years in controls	0.91 ± 0.12 mm	0.79 ± 0.13 mm	<0.001
Saxena et al. [22]	2017, Uttarakhand	163 ischemic stroke patients with a mean age of 58.43 ± 9.10 years	0.75 mm	0.56 mm	<0.0001
Kasliwal et al. [23]	2016, multicenter study: Delhi, Karnataka, Maharashtra, Madhya Pradesh, Telangana, Punjab, Kerala, Gujarat	1,157 persons aged ≥30 years and no previous CVD, the mean age of the subjects was 48.0 ± 12.0 years, and 54.2% were men	Not included	CIMT in men was 0.60 ± 0.12 mm, and in women was 0.57 ± 0.11 mm	<0.001
Das et al. [20]	2015, West Bengal	100 AIS patients, and 50 healthy controls, with a mean age in cases 57.21 ± 7.71 years and 54.7 ± 67.49 years in controls	0.84 ± 0.19 mm	0.60 ± 0.09 mm	<0.001
Sahoo et al. [19]	2009, Pondicherry	60 AIS patients and 50 healthy controls, with a mean age of 62 years, and 63% males in the case group	0.79 mm	0.60 mm	<0.0001

These observations demonstrate the fact that there were significant changes in the CIMT, probably attributed to lifestyles, dietary habits, and other sociocultural aspects specific to a particular geographic region, over the years.

Further highlighting the link between increased CIMT and cerebrovascular events, a previous study found a substantially larger CIMT in ischemic stroke patients compared to controls [19]. In a similar vein, Jain et al. [16] reported that the mean CIMT was 0.70 mm, ranging from 0.40 to 1.50 mm, in controls and 1.21 mm, with a range of 0.40-1.90 mm, in stroke patients. The difference closely matched the results of the current investigation and was statistically significant (p = 0.03).

The impact of gender on CIMT was assessed independently in both the control and case groups in this study. The control group's mean CIMT was 0.75 ± 0.35 mm for female patients and 0.93 ± 0.18 mm for male patients. Males had a greater mean CIMT, although this difference was not statistically significant (p = 0.21). This suggests that gender has no discernible impact on carotid wall thickness in people who have not experienced an AIS. The mean CIMT in the case group was 1.22 ± 0.19 mm in female patients and 1.08 ± 0.23 mm in men, as reported in a comparable analysis. Although CIMT levels were somewhat higher in female patients, the difference was not statistically significant (p = 0.22). Therefore, gender did not significantly affect CIMT in our study population, even among patients with AIS.

These results were in line with those of Juonala et al. [27], who noted a nonsignificant trend toward higher CIMT in male patients, highlighting the possibility that gender-related differences in CIMT may not always be apparent, particularly in studies with small sample sizes or closely matched cohorts. Similarly, research by Ahuja et al. [18] found that CIMT was considerably higher in stroke patients than in healthy people, regardless of gender. In contrast, Jain et al. [16] showed higher mean CIMT in female stroke patients (1.70 ± 0.30 mm) than in male patients (0.97 ± 0.40 mm). However, they noted that the study's very small number of female participants may have skewed the results. Notably, in the control group, male and female patients had the same mean CIMT (0.70 mm), indicating that there was no gender difference in the current control group.

The CIMT of stroke patients gradually increased with age, from 1.00 ± 0.00 mm in the 18-30 years age group to 1.21 ± 0.18 mm in those over 60 years, with an average of 1.12 ± 0.22 mm. The overall mean CIMT in the control group was 0.91 ± 0.19 mm. A significant variation in CIMT was noted across the 31-40 years (p = 0.0002), 41-50 years (p = 0.0476), and > 60 years (p < 0.0001) groups in both cases and controls. Moreover, the overall difference was statistically significant (p = 0.0007), demonstrating that age had a significant impact on CIMT in AIS and healthy controls in this study.

A previous study reported a substantial rise in CIMT across age groups among stroke patients (p < 0.05) [22]. Homma et al. [28] similarly discovered a consistent linear trend in CIMT with increasing age. Jain et al. [16] reported a consistent age-related increase in CIMT among cases, with values of 0.80 ± 0.30 mm in the 40-50 age group, 1.10 ± 0.40 mm in the 51-60 age group, and 1.30 ± 0.21 mm in the 61-70 age group. In contrast, control values were consistent throughout age groups. These findings lend credence to the notion that CIMT rises with age, particularly in individuals with cerebrovascular illness.

TLC (cases 8.89 ± 2.58 cells/mm^3^ vs. controls 12.78 ± 2.41 cells/mm^3^; t = 5.51, p < 0.0001) and Hb levels (cases 13.17 ± 2.47 g/dL vs. controls 8.49 ± 2.61 g/dL; t = -6.51, p < 0.0001) showed statistically significant differences between AIS patients and healthy individuals. Elevated Hb levels and lower TLC imply a possible change in hematology linked to stroke. The findings of the most recent Saudi Arabian investigation supported the link between Hb levels and ischemic stroke. This study's results showed that among AIS patients, higher Hb levels upon hospital admission are linked to more severe stroke outcomes [29]. Leukocytosis typically manifests in a number of situations, including inflammation, infection, and stress. While leukocytosis is widespread and linked to poor outcomes in AIS, leukopenia also occurs, as revealed by the results of this study, which demonstrated decreased TLC in cases than in the control group. This phenomenon can be explained by the fact that AIS causes a complex inflammatory response in the brain that results in post-stroke immunosuppression, infarct extension, secondary brain injury, and brain inflammation. There is evidence linking the hypothalamic-pituitary-adrenal axis to immunosuppression following a stroke, which may lead to extended glucocorticoid signaling and alter immunological responses [30].

There were no statistically significant differences between the groups in most parameters that were measured. LDL levels, however, came close to statistical significance (p = 0.05), indicating a possible trend that might become more evident in research with larger sample sizes. There was no discernible change in total cholesterol, TGs, or VLDL levels between stroke patients and controls, according to a similar study [18]. However, stroke patients had considerably higher LDL levels (115.67 mg/dL) than controls (102.26 mg/dL; p=0.0032) and significantly higher HDL levels (41.35 mg/dL) than controls (38.86 mg/dL; p=0.0408). These results imply that minor changes in HDL and LDL levels may be linked to a higher risk of stroke even in the absence of obvious lipid abnormalities.

Study limitations

With only 25 cases and 25 controls, the study's small sample size is a major limitation. This could limit the generalizability of the findings and the statistical power. The results might not be indicative of the general community because the study was only carried out at one tertiary-care facility, especially when it comes to people from varied geographic or healthcare settings. The study's capacity to demonstrate a causal link between elevated CIMT and the incidence of AIS is further limited by the cross-sectional case-control design, which only permits association conclusions. Furthermore, the lack of multivariate correction for confounders (e.g., smoking, diabetes, and HTN) reduces confidence in the obtained results. Patients with chronic stroke were not included in the study, which limited the evaluation of long-term vascular alterations and their possible contribution to the pathophysiology of stroke. The lack of ethnic and socioeconomic variety in the study population may restrict the results' external validity and applicability to a certain demographic group.

## Conclusions

In terms of CIMT, age but not gender distribution was among the demographic traits that differed significantly between the groups. Compared with controls, AIS patients had a significantly lower TLC and higher Hb levels. LDL levels were greater in AIS patients, indicating a possible correlation with ischemic events. Fasting blood glucose, HbA1c, and lipid profile did not differ significantly between cases and controls. The AIS patients' CIMT was substantially higher than that of the control group, suggesting a strong correlation between elevated CIMT and AIS. Although both groups' CIMT readings gradually increased with age, these changes were statistically significant in persons aged 30-40, 41-50, and >60 years. In conclusion, the study found that CIMT was markedly higher in patients with AIS, suggesting its role as a noninvasive and cost-effective marker for identifying individuals at high risk for cerebrovascular complications. Additionally, the available data indicate that CIMT values have been trending upward in both the case and control groups, with varying measurements observed across the nation. Large-scale investigations are needed to confirm these findings and assess the prognostic utility of CIMT across different populations. Further comprehensive population-level studies across the nation could validate the prevalence of CIMT measurements among healthy, diseased, and at-risk groups. This could increase awareness of CIMT, which may be the best noninvasive option for diagnosis, prognosis, and predicting AIS and possibly other vascular illnesses.
